# Correction: A new framework based on features modeling and ensemble learning to predict query performance

**DOI:** 10.1371/journal.pone.0300197

**Published:** 2024-03-04

**Authors:** Mohamed Zaghloul, Mofreh Salem, Amr Ali-Eldin

There is an error in the authors’ affiliation. The correct affiliation is: Computer Engineering and Control Systems Department, Faculty of Engineering, Mansoura University, Mansoura, Egypt.

## References

[1] Zaghloul M, Salem M, Ali-Eldin A (2021) A new framework based on features modeling and ensemble learning to predict query performance. PLoS ONE 16(10): e0258439. doi: 10.1371/journal.pone.0258439 34662344 PMC8523072

