# Supplementary material for: A new framework based on features modeling and ensemble learning to predict query performance
Source: PLoS One. 2021 Oct 18;16(10):e0258439. doi: 10.1371/journal.pone.0258439 (PMC8523072; doi:10.1371/journal.pone.0258439)
Supplement: Attachment — Submitted filename: Framework_FM_EL_Approach_202100807_V2_Response_Review.pdf [file pone.0258439.s002.pdf]

# A New Framework based on Features Modeling and Ensemble Learning to Predict Query Performance

Thank you to all of the reviewers who shared their thoughts. Please see the table below for responses to all reviewer comments.

## Reviewer #1:

| Comment No | Comment Description | Response |
|---|---|---|
| | | |
| 1. | Did the authors concentrate on optimization on joins and other complex queries such as nested sub queries in their experiment? | <u>*The following is mentioned in the training dataset metrics section home page 10. Queries syntax added to the supplementary material*</u><br><br>The queries which were used in the experiment were all complex, with sub queries and complex joins. The idea was to use complex queries from real-world implementations [the reference project was for a telecommunications company on a big data platform (cloudera environment) that handles streaming data]. The query syntax statement specifications, hardware specifications, software specifications, data architecture specifications, and historical performance logs will be used to derive these features. To extract input features from various types of queries, feature modeling is used.<br><br>An example of features extracted from syntax to extract query features.<br><br>1. Data_Transformation_No_Conditions<br>2. Data_Transformation_No_String_Conditions<br>3. Data_Transformation_No_Encoding_String_Conditions<br>4. Data_Transformation_No_Sub_Query<br>5. Data_Transformation_No_Agg<br>6. Data _Transformation_Frequency_Rate<br>7. Data _Transformation _No_Format_Func<br>8. Data_Transformation_Frequency_Rate<br>9. DT_Records_Number<br>10. Hour_of_the_data<br>11. Start_time<br>12. End_time |

<table>
<tr>
<td></td>
<td></td>
<td>
13. Day Performance<br><br>
The syntax of all queries used in the experiment work is attached and will be uploaded in the supplementary materials.<br><br>
Queries_Env.xlsx
</td>
</tr>
<tr>
<td>2.</td>
<td>It will be better for the readers if the author presents the parameters of the proposed NN model in a tabular column (epochs, architecture info and so on).</td>
<td>
<u>The following has been added to the “XGBoost Algorithm parameters section” home page 11</u><br><br>
XGBoost parameters<br>
<table>
<tr><th>Parameter</th><th>Parameter Value</th><th>Parameter Description</th></tr>
<tr><td>booster</td><td>gblinear</td><td>Select the type of model to run at each iteration. It has two options:<br><br>gbtree: tree-based models<br><br>gblinear: linear models</td></tr>
<tr><td>eta</td><td>0.0004</td><td>The learning rate used to weight each model</td></tr>
<tr><td>max_depth</td><td>10</td><td>The maximum depth of each tree</td></tr>
<tr><td>gamma</td><td>10</td><td>Gamma is a pseudo-regularization parameter</td></tr>
<tr><td>subsample</td><td>0.9</td><td>Represents the fraction of observations to be sampled for each tree</td></tr>
<tr><td>colsample_bytree</td><td>1</td><td>Number of features (columns) used in each tree</td></tr>
<tr><td>objective</td><td>“reg:squarederror"</td><td>reg:squarederror: for linear regression</td></tr>
<tr><td>eval_metric</td><td>"rmse"</td><td>rmse – root mean square error.</td></tr>
</table>
</td>
</tr>
</table>

| | | |
|---|---|---|
| | | |
| nrounds | 30000 | max number of boosting iterations. |
| nthreads | 10 | Number of threads can also be manually specified via |
| early_stopping_rounds | 20 | If NULL, the early stopping function is not triggered. If set to an integer k, training with a validation set will stop if the performance doesn't improve |
| verbose | 0 | If 0, xgboost will stay silent. If 1, it will print information about performance. If 2, some additional information will be printed out |
| | Results | The model performance measures the tested dataset that compared the predicted values versus actual values, the tested dataset being around 2723 records.<br>The mean absolute error (MAE) is given by.<br>$MAE = 1/N \sum_{i=1}^{n} \lvert y_i - \hat{y}_i \rvert$ = 0.663<br>(17)<br><br>The root mean square error (RMSE) is given by.<br>$RMSE = \sqrt{1/N \sum_{i=1}^{n} (y_i - \hat{y}_i)}$<br>= 0.8143<br>(18)<br>The average percentage difference between the actual and the predicted performance is |

| | | |
|---|---|---|
| | | equal to 5.3%, as shown in Fig 6. |
| 3. | What is the optimal value of 'k' in equation 7 that the authors have considered? | *<u>The following has been added to the "ensemble learning XGBoost algorithm" section home page 7</u>*<br><br>Xgboost takes a more iterative approach to determining the best K. The equation shows the iteration numbers that should be used in the XGBoost to achieve the smallest possible error. The number of rounds parameter is used as an input parameter in the XGBoost. The ensemble technique is based on a cleverer approach because it is based on many iterations that are combined to perform the final one. Rather than training all the models separately, boosting trains models that succeed, with each new model being trained to correct the mistakes made by the previous ones. Models are added in a sequential order until no further advancements can be made. So, rather than specifying an exact number, we provide the algorithm with a set of parameters to determine the optimal number of trees (no of estimators). |
| 4. | In the section "FEATURES ENGINEERING", the authors have discussed few pre-processing stages such as categorical data resolving and feature removal. Does the authors consider any feature selection algorithms such as filter methods, wrapper methods, for picking/dropping features? | *<u>The following has been added to the proposed framework section home page 5</u>*<br><br>The embedded features of the XGBoost are used in the proposed flow diagram. As a result, the feature engineering step is fed by the feed from feature importance. Label encoding also occurred. We proposed label encoding, which converts categorical features into numbers. The list of categorical features includes deployment environment encoded to (1 for cloud, 2 for on-premises), operating system environment encoded to (1 for Linux, 2 for Windows), data storage type encoded to (2 for SSD type), |

<table>
<tr>
<td></td>
<td></td>
<td>data transformation engine encoded to (1 for Hadoop, 2 for Oracle, and 3 for Greenplum), and query number encoded to 1 through 10 because we have 10 queries for data transformation.<br>
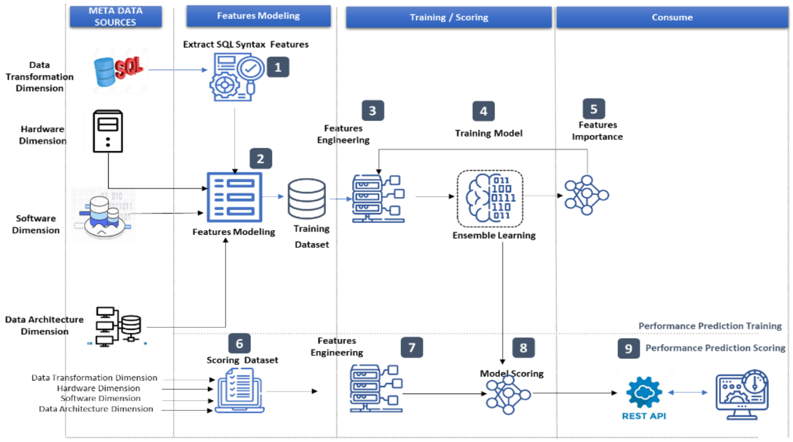
</td>
</tr>
<tr>
<td>5.</td>
<td>It is advised to compare the author's proposed work with any other standard work especially with the works discussed in section two (Related Work).</td>
<td><u>The following has been added to the discussion section home page 14</u><br><br>
DISCUSSION<br>
The key lessons of learning from the proposed approach and other approaches in the related work section, are shown in table 6. Number of features. Most of the approaches in the related work section limit the number of features used to train the prediction model to improve model accuracy. While the proposed approach did not limit the features, it did propose a generalization approach for features modelling to enrich the prediction model’s input training data set. The impact of feature standardization is that it standardizes the features used for training the prediction model despite differences in the query's environment specifications in terms of hardware and software, as well as data architecture. So, defining a feature domain, then extracting a list of features from each domain, generalizes an approach to standardizing the features. This step of standardizing the input features for the query prediction model is not considered in any of the related works. All the related work depends on the optimizer statistics to extract the logs and the features that impacted the query performance as per the historical execution plans. While the proposed approach standardized the input features considering the performance logs. Features Modelling is a step towards modelling the features, not to limiting the features. Other approaches just extract limited features. It is clear from the related work using the ensemble learning technique that the boosting methodology has shown better performance in such regression problems. A comparison between the related approach and the proposed approach is shown in table 7.</td>
</tr>
</table>

| Points | Classifier Approach | BOW Approach | KCCA Approach | Proposed Approach |
|---|---|---|---|---|
| Number of Features | Limited Features | Limited Features | Limited Features | No Limited Features |
| Features Standardization | NA | NA | NA | Features Modeling |
| Features Importance | NA | grid search | NA | Embedded in the ensemble learning |
| Machine Algorithm Approach | Classifier with any prediction algorithm | SVR, RFR and GBR | kernel canonical correlation analysis | XGBoost |
| Software Features Optimization | NA | NA | NA | Supported |
| Hardware Features Optimization | NA | NA | NA | Supported |
| Query Syntax Optimization | NA | NA | NA | Supported |
| Data Architecture Features Optimization | NA | NA | NA | Supported |

| | | |
|---|---|---|
| 6 | Please cite the following works to enhance the feature engineering-related works.<br><br>1. Ashokkumar P, Siva Shankar G, Gautam Srivastava, Praveen Kumar Reddy Maddikunta, and Thippa Reddy Gadekallu. 2021. A Two-stage Text Feature Selection Algorithm for Improving Text Classification. ACM Trans. Asian Low-Resour. Lang. Inf. Process. 20, 3, Article 49 (April 2021), 19 pages. DOI:https://doi.org/10.1145/3425781<br><br>2. G. Siva Shankar, P. Ashokkumar, R. Vinayakumar, Uttam Ghosh, | *Can be used in future work in automate query syntax feature extraction* |

| | | |
|---|---|---|
| | Wathiq Mansoor, Waleed S. Alnumay, "An Embedded-Based Weighted Feature Selection Algorithm for Classifying Web Document", Wireless Communications and Mobile Computing, vol. 2020, Article ID 8879054, 10 pages, 2020. https://doi.org/10.1155/2020/8879054 | |

# Reviewer #2:

| Comment No | Comment Description | Responses |
|---|---|---|
| 1 | The authors should emphasize the difference between other methods to clarify the position of this work further. | This has been answered responding to Reviewer-1 comment -5 |
| 2 | The Wide ranges of applications need to be addressed in Introduction | *The following is mentioned in the introduction section page 1*<br><br>Many factors influence query performance, such as data stores, data architecture, environment specifications, and query syntax. |
| 3 | The objective of the research should be clearly defined in the last paragraph of the introduction section | *The following is mentioned at the end of the introduction section home page 2.*<br><br>This paper aims to propose a query performance prediction framework that can be used to estimate query performance. The proposed framework can then be used as a query performance simulator to improve query syntax, optimize the allocated environment resources, and enhance the data architecture features used to execute the query. The main contribution is to model all the query features that influence query execution, feeding the ensemble learning technique with all the query-impactful features, to develop a query performance prediction model. The proposed framework can then be used as a query performance |

| | | |
|---|---|---|
| | | simulator to improve query syntax, optimize the allocated environment resources, and enhance the data architecture features used to execute the query. Section two discusses the related background, research motivation, and explains the challenges encountered while using the traditional approach, and sections three, four, and five introduce the proposed approach, experiment work, results, and discussion. Finally, section six summarizes this work. |
| 4 | Add the advantages of the proposed system in one quoted line for justifying the proposed approach in the Introduction section | *This has been answered responding to Reviewer-2 comment -3* |
| 5 | The motivation for the present research would be clearer, by providing a more direct link between the importance of choosing your own method. | *The following is added at the end of the related work section as per the reviewer's comment home page 5.*<br><br>The challenges of previous approaches, which relied solely on hardware specifications features such as (CPU, I/O, allocated RAM) to develop query performance prediction models, were highlighted in previous related work. The previously proposed models are not generalized enough to be used as a performance simulator to enhance query features that influence performance. Further, proposed hybrid models for determining which regression algorithm will fit into the distributed data space and produce the lowest error can be seen as a workaround. However, they are not optimal. As a consequence, we need to employ a more sophisticated regression algorithm. |
